# Dietary Pattern during 1991–2011 and Its Association with Cardio Metabolic Risks in Chinese Adults: The China Health and Nutrition Survey

**DOI:** 10.3390/nu9111218

**Published:** 2017-11-06

**Authors:** Ming Li, Zumin Shi

**Affiliations:** 1 Centre for Population Health Research, Division of Health Sciences, University of South Australia, City East Campus, North Tce, Adelaide, SA 5001, Australia; 2 Discipline of Medicine, University of Adelaide, Adelaide, SA 5005, Australia; Zumin.shi@adelaide.edu.au

**Keywords:** The China Health and Nutrition Survey, dietary pattern, principal component analysis, cardio-metabolic risks, adults

## Abstract

Increased prevalence of overweight and obesity, diabetes, hypertension, and other cardio metabolic risks has become a public health concern in China, a country undergoing nutrition transition. We investigated the dietary pattern during 1991–2011 and its association with these risks in a longitudinal study among adults; Adults in The China Health and Nutrition Survey were included. Three-day food consumption was collected by 24 h recall method. Anthropometric measures, blood pressure, fasting blood glucose and lipids was collected in 2009. Dietary pattern was generated using principal components analysis. The associations between dietary pattern and cardio metabolic risk were assessed with generalized linear regression adjusted for age, sex, and social economic status (SES). “Traditional” pattern loaded with rice, meat, and vegetables, and “Modern” pattern had high loadings of fast food, milk, and deep-fried food. “Traditional” pattern was inversely associated with cardio metabolic risks, with linear slopes ranging from −0.15 (95% confidence interval (CI): −0.18, −0.12) for hypertension to −0.67 (95% CI: −0.73, −0.60) for impaired glucose control. “Modern” pattern was associated positively with those factors, with slopes ranging 0.10 (95% CI: 0.04, 0.17) for high cholesterol to 0.42 (95% CI: 0.35, 0.49) for impaired glucose control. Dietary patterns were associated with cardio metabolic risk in Chinese adults.

## 1. Introduction

In the past three decades, China has undergone dramatic economic and environmental development, both of which impact greatly on daily life and health of each individual. Among the various dimensions of change, the most outstanding one has been “nutrition transition”, the transition from traditional diets high in cereal and fiber to more Western pattern diets high in sugars, fat, and animal-source foods that is happening as in low- and middle-income countries [1,2,3]. The China Health and Nutrition Survey (CHNS) during 1991–2009 has shown the shift in the form of either nutrients or food items or dietary patterns and this dietary shift is associated with education, income, urbanicity, and macro food environment and policy [1,2,4,5,6,7]. Concurrently with the dietary shift, the health status of the population has also shifted to a pandemic of obesity and increased cardio metabolic risks [8]. A study using anthropometric and fasting blood measures in 2009 CHNS reported that 30% Chinese adults had body mass index (BMI) ≥ 25 kg/m^2^, and that more than two-thirds had at least one of the cardio metabolic risks including pre-diabetes/diabetes, hypertension, dyslipidemia, and high C-creative protein [9]. These expanding burdens of cardio metabolic risks were more prevalent in less urbanized areas at all income levels [9]. Higher prevalence of the cardio metabolic risks was associated with diet and physical inactivity and overweight/obesity in a cross-sectional analysis using the CHNS data [10].

Dietary pattern generated from principal component analysis in CHNS before 2009 waves was found to be associated with overweight, hypertension, or chronic kidney diseases in adults or in older populations [11,12,13]. The most recent food intake collected from the 2011 survey is available [14] and the association with cardio metabolic risks has not been investigated besides overweight/obesity and hypertension [2]. Here we report the dietary pattern in all the waves during 1991–2011 and its association with all cardio metabolic risks including overweight/obesity, hypertension, diabetes, dyslipidemia, impaired glucose control, and metabolic syndrome in adults. We hypothesize that the traditional dietary pattern was negatively associated with cardio metabolic risks, while the modern dietary pattern was positively associated with cardio metabolic risks.

## 2. Materials and Methods

### 2.1. Study Population

The CHNS was an ongoing open cohort longitudinal survey of nine waves (1989–2011). A multistage, random cluster process was used to obtain the sample in nine provinces across China varying substantially in geography, economic status, and health status—detailed CHNS sampling and cohort profile information are described elsewhere [14,15]. Survey protocols, instruments, and the process of obtaining informed consent for CHNS were approved by the institutional review committees of the University of North Carolina at Chapel Hill and the National Institute of Nutritional and Food Safety, China Centre for Disease Control and Prevention [14,15]. We included eight waves of data collected from all adults aged over 18 years in this study in 1991, 1993, 1997, 2000, 2004, 2006, 2009, and 2011.

### 2.2. Study Outcome

Cardio metabolic risks included in this study were overweight/obesity, hypertension, and diabetes, high cholesterol, triglycerides, low density lipoprotein (LDL), and low high density lipoprotein (HDL), impaired glucose control, and metabolic syndrome. They were derived from anthropometric measures or fasting blood tests in 2009. The definitions of the risks are listed in Table 1.

Height was measured to 0.1 cm without shoes using SECA stadiometer, weight was recorded to the nearest 0.1 kg with light cloth on a calibrated beam scale. Systolic and diastolic blood pressure (SBP and DBP) were recorded as means of three reads after 10 min of rest using mercury sphygmomanometers with appropriated cuff sizes. All the measures were taken using World Health Organization (WHO) standard protocol by trained staff.

Fasting blood was taken early in the morning and prepared for further testing in a national central lab in Beijing (medical laboratory accreditation certificate ISO 15189: 2007) in 8657 adults. Fasting glucose was measured with the GOD-PAP (glucose oxidase-phenol plus aminophenazone) method (Randox Laboratories Ltd., Crumlin, UK), HbA1C was tested using high performance liquid chromatography system (HLC-723 G7; Tosoh Corporation, Tokyo, Japan). Fasting lipids were tested with Hitachi 7600 automated analyzer (Hitachi Inc., Tokyo, Japan) with determiner regents (Kyowa Medex Co., Ltd., Tokyo, Japan). All the measurements and tests were collected using standard protocol by trained staff. The detailed data collection protocol has been described elsewhere [9,14,15].

### 2.3. Dietary Assessment and Dietary Patterns

The food intake for the eight waves of the CHNS involves three consecutive 24 h recalls at the individual level and a household inventory in the same period randomly allocated during a week. Food consumption including the type and amount of food and meal was collected by a trained investigator. Cooking oil and condiments for each individual in the household was estimated using the household inventory. A detailed description of the dietary assessment has been published [4].

Thirty-five food groups were generated based on similar nutrient profiles or culinary uses which are similar to the food items used in the 2002 Chinese national nutrition survey [11]. 

Dietary patterns (main independent variables) across the eight waves (1991–2011) were identified by factor analysis using the standard principal component analysis method. Patterns were determined by eigenvalue (>1), scree plot, and factor interpretability by each factor. Labelling of the patterns was primarily descriptive and based on our interpretation of the pattern structures.

Factor loadings are equivalent to simple correlations between the food items and the patterns. Higher loadings (absolute value) indicate that the food shares more variance with that pattern. The sign of the loading determines the direction of the relationship of each food to the factor. Factor loadings were graphically presented.

At each wave, participants were assigned pattern-specific factor scores. Scores for each pattern were calculated as the sum of the products of the factor loading coefficients and standardized daily intake of each food group associated with that pattern. Cumulative scores were added across seven waves (1991–2009), a cumulative mean score was calculated for each pattern, as it better reflects long-term diet and may reduce dietary measurement error and has been used in other studies [11,16].

### 2.4. Covariates

Detailed information on sociodemographic and lifestyle factors was collected at each wave using a structured questionnaire. Urbanization is defined by a twelve-component urbanization index including factors of population density, physical, social, cultural, and economic environments [5]. The urbanization index was recoded into tertiles. Education was grouped into three categories (low: illiterate/primary school; medium: junior middle school; and high: high middle school or higher) using highest education level in the survey questionnaire. Per capita annual family income was recoded into tertiles (low, medium, and high). Smoking status was categorized as non-smokers, ex-smokers, and current smokers. Physical activity level (metabolic equivalent of task (MET)) was estimated based on self-reported activities (including occupational, domestic, transportation, and leisure time physical activity) and duration using a Compendium of Physical Activities [17]. Alcohol consumption was allocated to five categories as “None, <1/week, 1–2/week, 3–4/week, and daily”.

### 2.5. Data Analysis

Basic characteristics of the study population were summarized by waves and cardio metabolic risks. The mean cumulative score of each dietary pattern was compared by categories of the cardio metabolic risks using t test. The association between the cardio metabolic risks and dietary patterns was assessed using a general linear regression model and adjusted for age and sex in “Model 1”. Socioeconomic factors (urbanization, household income, and education) were further adjusted in “Model 2”. “Model 3” was the final adjusted model by additionally including smoking, drinking, and physical activity. A sensitivity analysis was conducted among adults having all of the seven waves of diet assessment and measured cardio metabolic risks (*n* = 2318). All the analyses were performed by suing STATA 13.1 (STATA Corporation, College Station, TX, USA). Statistical significance was considered when *p* < 0.05.

## 3. Results

During the 1991–2011 surveys, the mean age of the sample increased substantially and the proportion of female participants increased. Significantly more participants in the later waves fell in higher urbanization and education categories but not household income. The mean BMI, SBP, DBP were significantly higher in later waves while the proportions of non-smokers, non-drinkers were lower compared to the earlier waves (see Table S1 in the supplementary materials).

In 2009, 29.4% of the participants had a BMI ≥ 25 kg/m^2^ and the prevalence of abdominal overweight/obesity was 37.3%. A total of 2576 (26.9%) were classified as having hypertension. Nine hundred and sixty (960) participants (11.1%) had diabetes and 3280 out of 8611 were defined as having impaired glucose control (HbA1C ≥ 5.7%). The prevalence of high triglycerides, cholesterol, and LDL was 24.5%, 34.2%, and 30.6%, respectively, and 2256 in 8656 (26.1%) had low HDL. There were 1890 participants among 9465 (20.0%) who had metabolic syndrome. The characteristics of participants having the cardio metabolic risks are presented in Table 2. Higher prevalence of cardio metabolic risk was more likely in older participants, or those living in highly urbanized areas, or having higher household income, or having lower education level. Those with overweight/obese, hypertension, diabetes, metabolic syndrome, or dyslipidemia were more likely current smokers or drinkers, or more likely to have lower physical activity levels. Abdominal overweight/obesity, higher LDL or total cholesterol, or metabolic syndrome was more prevalent in women, while hypertension, diabetes, higher triglycerides, or low HDL was more common in men. No gender difference was found for BMI or HbA1C.

**Figure 1:**
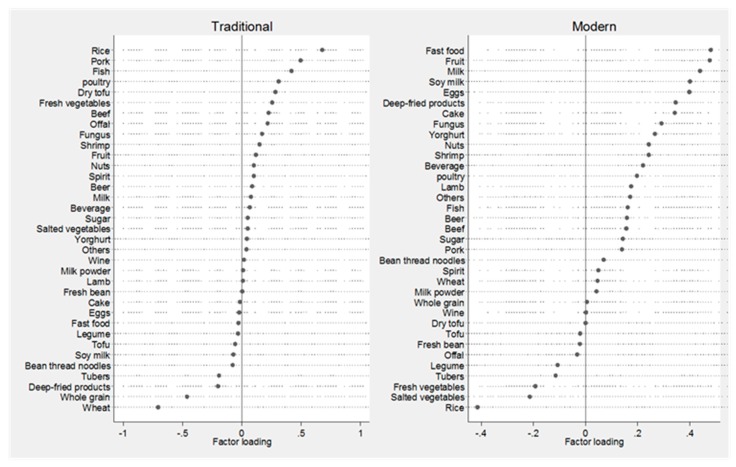
Dietary pattern loading by 35 food groups during 1991–2011.

Two dietary patterns were constructed explaining 12% variance in the food intake. The first pattern was positively loaded for food groups of rice, pork, fish, poultry, tofu, and vegetables and negatively load with wheat, whole grains, and deep-fried food. This pattern was named “Traditional”, while the second pattern was positively loaded for groups of fast food, milk, deep-fried food, and cake and negatively loaded for rice, vegetables, and tubes. This pattern was named “Modern”. The dietary patterns with food group loading are presented in Figure 1.

**Figure 2:**
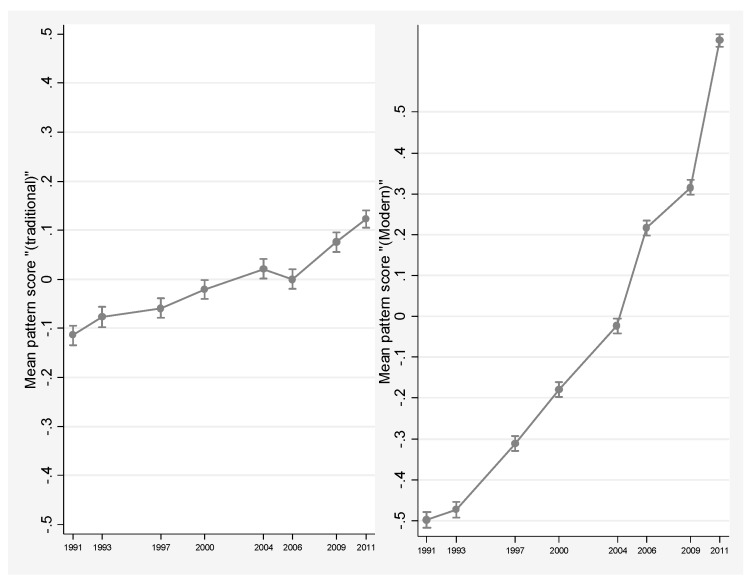
Age- and sex-adjusted mean dietary pattern scores during 1991–2011.

Figure 2 shows the trend of mean scores in the eight waves during 1991–2011. The “Traditional” pattern remained relatively stable, but a dynamic trend and sharp increase is seen in the “Modern” pattern. Both dietary patterns were associated with urbanization and household income. Specifically, the mean age- and sex-adjusted dietary pattern scores were significantly higher in participants from highly urbanized and educated households (see Figure S1).

The cumulative mean score of both dietary patterns in seven waves during 1991–2009 was compared by cardio metabolic risks and is presented in Table 3. The “Traditional” score was significantly lower in those with overweight/obesity, hypertension, diabetes, low HDL, metabolic syndrome, and impaired glucose control compared with the corresponding counterpart. The score seemed higher in those with high triglycerides and cholesterols (*p* < 0.001). The “Traditional” pattern score was not different according to LDL categories. On the other hand, the cumulative mean “Modern” pattern scores were consistently higher among participants with overweight/obesity, diabetes, high triglycerides or cholesterol, or LDL, low HDL, metabolic syndrome, and impaired glucose control, but the “Modern” dietary pattern score did not differ by hypertension status in this population.

The multivariable adjusted association between dietary patterns and cardio metabolic risks is presented in the Table 4. Overall, the “Traditional” pattern was negatively associated with overweight/obesity, hypertension, diabetes, metabolic syndrome, and impaired glucose control, with the beta coefficient ranging from −0.15 (95% CI: −0.18, −0.12) for hypertension to −0.67 (95% CI: −0.73, −0.60) for impaired glucose control but not consistent for blood lipid risks. On the other hand, the “Modern” pattern was positively associated with beta coefficients ranging from 0.10 (95% CI: 0.04, 0.17) for high cholesterol to 0.42 (95% CI: 0.35, 0.49) for impaired glucose control. The “Modern” pattern was not significantly associated with triglycerides and HDL. The associations between both dietary patterns and cardio metabolic risks were independent of demographic, socioeconomic, and lifestyle factors and not modified by age or sex. Sensitivity analysis did not show any significance change of the corresponding association but seemed to intensify the association, particularly for the “Modern” pattern, such that the adjusted beta coefficient for overweight/obesity was 0.18 (95% CI: 0.11, 0.24) in participants having at least one diet assessment compared to a coefficient of 0.67 (95% CI: 0.58, 0.77) in participants having all waves of dietary assessment.

## 4. Discussion

Using the most recent data from all waves of CNHS during 1991–2011, we studied dietary pattern and its prospective association with cardio metabolic risks including overweight/obesity, hypertension, diabetes, dyslipidemia, metabolic syndrome, and impaired glucose control in the general Chinese adult population.

By including dietary intake in 2011, we confirmed two dietary patterns that were reported in other studies using earlier survey waves [4,11,12,13]. The “Traditional” pattern was positively correlated with rice, vegetables, and meat and the “Modern” pattern was positively correlated with fast food, and fried products, cakes, and milk. These results indicated a dramatic change in dietary pattern in the past two decades, especially the steep increase of the “Modern” pattern, while the “Traditional” pattern was stable over the study period. We found that urbanicity, household income, and education were associated with higher scores, thus suggesting higher food variety for people with high income and education living in urban areas. This dietary pattern trend may reflect the shift from a traditional agriculture system to a modern business system with the rapid growth of retail and food service sectors appearing in the past decades. The system shift brought about diet change towards more savory processed food supplies with refined carbohydrates, added salt and sweetener, edible oils, animal-resource foods, and decreased intake in legumes, fresh fruit, and vegetables [18]. Foods are becoming easy to access in neighborhoods and eating away from home is becoming more popular in urban areas, as more catering services are increasingly becoming available, all of which has been associated with weight status in China [5,19]. It is important to develop effective strategies to cope with the system and environmental changes to ensure people are equipped with adequate information and knowledge to choose healthy products from a well-managed food market.

This study showed that the “Modern” dietary pattern was positively associated with cardio metabolic risks such as obesity, hypertension, impaired glucose control/diabetes, and metabolic syndrome, which have consistently been reported in cross-sectional or longitudinal studies in different populations [8,10,12,13,14]. The “Modern” dietary pattern was positively correlated with high fat, high salt foods, such as fried fast foods, and the association with overweight and obesity, insulin resistance, and hypertension has been well established [20,21,22]. This emerging dietary pattern, which has a tendency of first hitting people with higher incomes and/or residing in urban areas, and its association with the growing prevalence of cardio metabolic risks should be a concern that calls for large-scale and extensive strategies across all sectors including government, food supply, food environment, education, and public health. This is a major challenge in China and developing countries undergoing nutrition transition, as few are serious in addressing prevention of the dietary shift in spite of some large-scale programmatic and policy shift exploration in South American countries [1]. On the other hand, our results showed that the “Traditional” pattern was persisting and protective from cardio metabolic risks including overweight/obesity, as consistently reported in other studies [8,10,12,13,14]. The body of evidence can back up the possible merits of traditional dietary practices in facing nutrient transitions. In South Korea, government, nutrition specialists, and some private organizations have worked to retain healthful aspects of the traditional diet based on the evidence that traditional diets were protective from having obesity, and contributed to low levels of total fat in the overall diet and high intakes of fruit and vegetables (a large proportion of which comes from Kimchi) in South Korea [23]. Continuing national-wide diet monitoring, food policy, and regulation has been carried out to promote healthy eating habits in addition to the construction of healthy environments by the creation of laws and programs and by research and social marketing [24].

There was no clear association between the “Traditional” pattern and blood lipids, while the “Modern” pattern was associated with high LDL and high cholesterol. This lack of a definite association could be attributable to the net effect of food groups in both dietary patterns, and the complicated metabolic mechanism of lipoprotein, which warrants further investigation. For example, rice, as a principal component in the “Traditional” diet pattern, has been positively associated with abnormal high-density lipoprotein but not high triglycerides in Chinese adults [25], while rice intake was negatively related to total cholesterol level in the Japanese population [26].

The major strength of the CHNS is the ability to capture enormous heterogeneity and spatial and temporal change in one of the most rapidly changing environments in the world. Because of its long duration and wide geographic coverage, the CHNS can document the dramatic economic, social, behavioral, and health status changes that have characterized China in the past several decades [13]. The prospective association was achieved by using dietary patterns from 1991 to 2009 and objectively collected anthropometric and biochemical measures from fasting blood samples in 2009. Cumulative dietary patterns from multiple rounds of food intake by three-day recalls in all adults may have attenuated misclassification of dietary patterns arising from intake collected by a single three-day recall. The association between dietary and cardio metabolic risks was adjusted for a variety of socioeconomic and lifestyle factors. The association was robust, as shown by sensitivity analysis and additional adjusting for variation of covariates during the study period by mixed effect modeling (e.g., age, BMI, social economic status, smoking, drinking, physical activity). Limitations of the study should be noted. Firstly, the prevalence of cardio metabolic risks could be biased because the blood samples were collected from volunteers in the survey in 2009, though the association with diet pattern was not affected. Secondly, we were unable to explore the association of diet with other biomarkers such as alanine aminotransferase and C-reactive protein collected in 2009 due to the lack of detailed disease and medication information needed to tease out important confounders in the association.

In summary, two dietary patterns named “Traditional” and “Modern” were identified using eight waves of food intake data from the CHNS in the past two decades. The “Traditional” pattern was loaded with rice, meat, and vegetables and remained stable and persistent. This pattern was protective from having a range of cardio metabolic risks including overweight/obesity, hypertension, diabetes, and metabolic syndrome. The “Modern” pattern, loaded with fast food, milk, cakes, and deep-fried foods, was consumed by increasingly more people, especially those living in more urbanized areas or having higher education levels. The “Modern” pattern was associated with a higher prevalence of the cardio metabolic risks. 

## Figures and Tables

**Table 1:** Definitions of cardio metabolic risks.

Cardio Metabolic Risks	Definition
Overweight/obesity	Body mass index (BMI) ≥ 25 kg/m^2^
Abdominal overweight/obesity	Waist circumference (WC) ≥ 94 cm in males or ≥80 cm in females
Hypertension	Systolic blood pressure (SBP) ≥ 140 mmHg and/or diastolic blood pressure (DBP) ≥ 90 mmHg or have known hypertension
Diabetes	Fasting plasm glucose > 7.0 mmol/L, or having known diabetes
High cholesterol	Cholesterol ≥ 200 mg/dL
High triglycerides	Triglycerides ≥ 150 mg/dL
High LDL	low density lipoprotein (LDL) > 130 mg/dL
Low HDL	high density lipoprotein (HDL) < 49 mg/dL in males or <50 mg/dL in females
Impaired glucose control	HbA1C ≥ 5.7%
Metabolic syndrome	WC ≥ 90 cm in males or WC ≥ 80 cm in females plus two or more of the following: Raised triglycerides as triglycerides ≥ 150.6 mg/dL; reduced HDL as HDL < 39.8 mg/dL in males or <49.9 mg/dL in females; raised blood pressure as systolic ≥ 130 mmHg or diastolic ≥ 85 mmHg or treated of hypertension, raised plasma glucose as glucose ≥ 5.6 mmol/L

**Table 2:** Cardio metabolic risks collected in the 2009 round of the China Health and Nutrition Survey.

	BMI ≥ 25 (kg/m^2^)	Abdominal Overweight/Obesity	Hypertension	Diabetes	High Triglycerides	Low HDL	High LDL	High Cholesterol	Metabolic Syndrome	Impaired Glucose Control
*n*/*N*	2788/9499	3523/9440	2576/9586	960/8658	2113/8625	2256/8656	2649/8654	2950/8625	1890/9465	3280/8611
**Age (years)**	51.9 (13.5)	53.7 (14.1)	60.0 (12.9)	59.3 (12.8)	51.9 (13.8)	50.5 (14.6)	54.9 (13.7)	54.7 (13.6)	56.3 (12.8)	56.0 (13.9)
**Male (%)**	1321 (47.4)	825 (23.4)	1288 (49.9)	482 (50.0)	1102 (52.0)	716 (31.7)	1127 (42.5)	1289 (43.6)	690 (36.5)	1551 (47.3)
**Urbanization**										
Low	344 (12.3)	495 (14.0)	328 (12.7)	119 (12.3)	251 (11.8)	322 (14.27)	304 (11.5)	342 (11.6)	235 (12.4)	585 (17.8)
Medium	945 (33.9)	1217 (34.5)	869 (33.7)	258 (26.8)	698 (32.9)	735 (32.6)	837 (31.6)	951 (32.2)	596 (31.5)	1018 (31.0)
High	1499 (53.8)	1821 (51.5)	1382 (53.6)	587 (60.9)	1171 (55.2)	1199 (53.2)	1508 (56.9)	1665 (56.3)	1059 (56.0)	1677 (51.1)
**Income**										
Low	700 (25.4)	941 (27.0)	711 (28.0)	250 (26.4)	548 (26.3)	656 (29.6)	742 (28.4)	818 (28.0)	480 (26.0)	892 (27.6)
Medium	854 (31.0)	1083 (31.1)	760 (29.9)	268 (28.3)	664 (31.9)	683 (30.8)	801 (30.7)	926 (31.7)	539 (29.1)	969 (22.9)
High	1199 (43.6)	1460 (41.9)	1068 (42.1)	428 (45.2)	872 (41.8)	876 (39.6)	1068 (40.9)	1173 (40.2)	831 (44.9)	1376 (42.5)
**Education**										
Low	1143 (41.1)	1744 (49.4)	1384 (53.8)	486 (50.6)	862 (40.8)	991 (44.0)	1160 (43.9)	1327 (45.0)	949 (50.2)	1557 (47.6)
Medium	965 (34.7)	1083 (30.7)	699 (27.2)	266 (27.7)	694 (32.8)	720 (32.0)	833 (31.5)	910 (30.9)	569 (30.1)	1004 (30.7)
High	676 (24.3)	704 (19.9)	489 (19.0)	209 (20.6)	559 (26.4)	539 (24.0)	648 (64.5)	710 (24.1)	371 (19.6)	712 (21.8)
**BMI (Kg/m^2^)**	27.6 (2.2)	25.7 (3.3)	24.7 (3.7)	25.3 (3.9)	25.1 (3.5)	24.7 (3.6)	24.1 (3.4)	24.1 (3.4)	26.4 (3.3)	24.4 (3.6)
**SBP (mmHg)**	131.2 (19.4)	130.1 (20.4)	146.4 (18.2)	134.8 (19.4)	129.4 (19.6)	126.4 (19.7)	128.5 (19.1)	128.8 (19.2)	136.8 (20.0)	129.8 (19.6)
**DBP(mmHg)**	84.8 (11.3)	80.7 (10.3)	91.7 (11.6)	84.5 (11.3)	83.6 (11.3)	81.7 (11.6)	82.3 (11.2)	82.6 (11.3)	86.5 (11.4)	82.8 (11.3)
**Smoking**										
Never	1988 (71.4)	2952 (83.6)	1734 (67.3)	664 (69.0)	1387 (65.6)	1742(77.4)	1882 (71.2)	2085 (70.7)	1416 (75.0)	2221 (67.8)
Previous	109 (3.9)	74 (2.1)	141 (5.5)	45 (4.7)	72 (3.4)	60 (2.7)	86 (3.3)	90 (3.1)	69 (3.7)	129 (3.9)
Current	689 (24.7)	506 (14.3)	703 (27.3)	253 (26.3)	657 (31.1)	449 (20.0)	675 (25.5)	776 (26.3)	403 (21.4)	925 (28.2)
**Drinking**										
Never	1845 (66.5)	2794 (79.4)	1758 (68.4)	658 (68.6)	1340 (63.7)	1744 (77.8)	1827 (69.6)	1991 (67.8)	1389 (73.8)	2218 (68.1)
<1/week	313 (12.3)	260 (7.4)	228 (8.9)	87 (9.1)	242 (11.5)	225 (10.0)	257 (9.8)	279 (9.5)	162 (8.6)	326 (10.0)
1–2/week	226 (8.1)	168 (4.8)	184 (7.2)	73 (7.6)	191 (9.1)	115 (5.1)	157 (7.5)	231 (7.9)	117 (6.2)	245 (7.5)
3–4/week	129 (4.7)	102 (2.9)	82 (3.2)	33 (3.4)	107 (5.1)	56 (2.5)	89 (3.4)	108 (3.7)	60 (3.2)	130 (4.0)
Daily	263 (9.5)	197 (5.6)	318 (12.4)	103(11.6)	224 (10.7)	102 (4.6)	257 (9.8)	327 (11.1)	154 (8.2)	339 (10.4)
**Median METs (IQR)**	90.9 (30.0, 182.0)	90.3 (31.5, 147.6)	97.2 (33.0, 190.3)	142.8 (31.0, 57.9)	98.5 (36.9, 178.8)	98.5 (36.9, 178.3)	98.5 (36.9, 178.3)	67.4 (33.5, 158.4)	90.3 (31.5, 174.6)	68.2 (33.0, 168.3)

*N*: the number of participants with available cardio metabolic results; Abbreviations: BMI: body mass index; LDL: low density lipoprotein; HDL: high density lipoprotein; SBP: systolic blood pressure; DBP: diastolic blood pressure; METs: metabolic equivalent task score; IQR; interquartile range; Urbanization defined by a twelve-component urbanization index including capture population density and physical, social, cultural, and economic environments [5]; Income defined by per capita annual family income; Abdominal overweight/obesity defined as WC ≥ 94 cm in males and ≥80 cm in females; Hypertension defined as SBP ≥ 140 mmHg and/or DBP ≥ 90 mmHg and/or previously diagnosed hypertension; Diabetes defined as fasting plasm glucose > 7.0 mmol/L and/or previously diagnosed diabetes; High cholesterol defined as cholesterol ≥ 200 mg/dL; high triglycerides as triglycerides ≥ 150 mg/dL; low HDL as HDL < 40 mg/dL in males or <50 mg/dL in females; high LDL as LDL ≥ 130 mg/dL; Impaired glucose control defined as HbA1C ≥ 5.7%; Metabolic syndrome defined by International Diabetes Federation (IDF) criteria using waist circumference, triglycerides, HDL, blood pressure and plasma glucose as indicators.

**Table 3:** Mean cumulative pattern score (1991–2009) by cardio metabolic risks in 2009 among adults in the China Health and Nutrition Survey (CHNS).

Risk Categories	No.	Traditional (95% CI)	*p*	Modern (95% CI)	*p*
BMI ≥ 25 kg/m^2^			<0.001		<0.001
No	6693	0.05 (0.03, 0.07)		0.03 (0.01, 0.05)	
Yes	2781	−0.10 (−0.013, −0.07)		0.16 (0.13, 0.19)	
Abdominal overweight/obesity			<0.001		<0.001
No	5917	0.09 (0.07, 0.11)		0.03 (0.01, 0.05)	
Yes	3523	−0.13 (−0.16, −0.10)		0.12 (0.09, 0.15)	
Hypertension			<0.001		0.47
No	7010	0.02 (0.001, 0.04)		0.07 (0.05, 0.09)	
Yes	2576	−0.05 (−0.08, −0.01)		0.05 (0.02, 0.08)	
Diabetes			<0.001		<0.001
No	7698	0.03 (0.01, 0.05)		0.04 (0.02, 0.06)	
Yes	960	−0.13 (−0.19, −0.08)		0.18 (0.13, 0.23)	
High triglycerides			<0.001		<0.001
No	6512	−0.0001 (−0.02, 0.02)		0.03 (0.01, 0.05)	
Yes	2113	0.06 (0.02, 0.10)		0.12 (0.09, 0.16)	
High cholesterol			0.03		<0.001
No	5675	0.001 (−0.02, 0.02)		0.02 (0.001, 0.04)	
Yes	2950	0.04 (0.01, 0.07)		0.11 (0.08, 0.14)	
High LDL			0.98		<0.001
No	5978	0.01 (−0.01, 0.04)		0.02 (0.001, 0.04)	
Yes	2644	0.01 (−0.02, 0.05)		0.12 (0.09, 0.15)	
Low HDL			0.002		0.02
No	6378	0.03 (0.01, 0.05)		0.04 (0.02, 0.06)	
Yes	2246	−0.03 (−0.07, 0.003)		0.09 (0.05, 0.12)	
Metabolic syndrome			<0.001		<0.001
No	7555	0.03 (0.01, 0.05)		0.05 (0.03, 0.06)	
Yes	1855	−0.09 (−0.13, −0.05)		0.13 (0.10, 0.17)	
Impaired glucose control			<0.001		<0.001
No	5319	0.19 (0.17, 0.21)		−0.01 (−0.03, 0.01)	
Yes	3271	−0.27 (−0.30, −0.24)		0.15 (0.12, 0.18)	

Abbreviations: CI: confidence interval; BMI: body mass index; LDL: low density lipoprotein; HDL: high density lipoprotein; Abdominal overweight/obesity defined as WC ≥ 94 cm in males and ≥80 cm in females; hypertension defined as SBP ≥ 140 mmHg and/or DBP ≥ 90 mmHg and/or previously diagnosed hypertension; Diabetes defined as fasting plasm glucose > 7.0 mmol/L and/or previously diagnosed diabetes; High cholesterol defined as cholesterol ≥ 200 mg/dL; high triglycerides as triglycerides ≥ 150 mg/dL; low HDL as HDL < 40 mg/dL in males or <50 mg/dL in females; high LDL as LDL ≥ 130 mg/dL; Impaired glucose control defined as HbA1C ≥ 5.7%; Metabolic syndrome defined by IDF criteria using waist circumference, triglycerides, HDL, blood pressure, and plasma glucose as indicators.

**Table 4:** Association (beta coef.) between dietary pattern score during 1991–2009 and cardio metabolic profiles in adults in 2009.

	BMI ≥ 25 (kg/m^2^)	Abdominal Overweight/Obesity	Hypertension	Diabetes	High Triglycerides	Low HDL	High LDL	High Cholesterol	Metabolic Syndrome	Impaired Glucose Control
Traditional pattern										
Crude	−0.20 (−0.22, −0.18)	−0.29 (−0.34, −0.24)	−0.11 (−0.13, −0.09)	−0.21 (−0.28, −0.13)	0.08 (0.02, 0.13)	−0.08 (−0.14, −0.03)	0.001 (−0.05, 0.05)	0.05 (−0.001, 0.10)	−0.15 (−0.21, −0.10)	−0.61 (−0.67, −0.056)
Model 1	−0.18 (−0.21, −0.16)	−0.27 (−0.33, −0.22)	−0.06 (−0.08, −.003)	−0.20 (−0.27, −0.12)	0.07 (0.01, 0.13)	−0.05 (−0.11, 0.01)	0.04 (−0.02, 0.09)	0.09 (0.03, 0.14)	−0.13 (−0.19, −0.07)	−0.64 (−0.69, −0.58)
Model 2	−0.35 (−0.37, −0.33)	−0.32 (−0.38, −0.26)	−0.14 (−0.17, −0.11)	−0.29 (−0.37, −0.21)	0.02 (−0.04, 0.08)	−0.07 (−0.13, −0.01)	−0.03 (−0.09, 0.02)	0.02 (−0.03, 0.08)	−0.19 (−0.25, −0.12)	−0.68 (−0.74, −0.62)
Model 3	−0.35 (−0.37, −0.32)	−0.33 (−0.39, −0.27)	−0.15 (−0.18, −0.12)	−0.24 (−0.33, −0.15)	0.03 (−0.04, 0.09)	−0.04 (−0.11, 0.02)	−0.04 (−0.10, 0.02)	0.01 (−0.05, 0.07)	−0.18 (−0.25, −0.12)	−0.67 (−0.73, −0.60)
Sensitivity analysis	−0.45 (−0.50, −0.40)	−0.35 (−0.46, −0.23)	−0.11 (−0.16, −0.05)	−0.21 (−0.36, −0.05)	0.08 (−0.04, 0.20)	−0.05 (−0.18, 0.07)	−0.04 (−0.15, 0.06)	0.04 (−0.06, 0.14)	−0.20 (−0.32, −0.07)	−0.73 (−0.84, −0.62)
Modern pattern										
Crude	0.19 (0.13, 0.24)	0.13 (0.08, 0.18)	−0.02 (−0.08, 0.03)	0.19 (0.11, 0.26)	0.13 (0.07, 0.19)	0.07 (0.01, 0.13)	0.15 (0.09, 0.20)	0.13 (0.08, 0.18)	012 (0.06, 0.18)	0.24 (0.18, 0.29)
Model 1	0.21 (0.16, 0.26)	0.25 (0.19, 0.30)	0.09 (0.03, 0.15)	0.27 (0.19, 0.34)	0.14 (0.09, 0.20)	0.08 (0.02, 0.14)	0.21 (0.16, 0.27)	0.20 (0.14, 0.25)	0.20 (0.14, 0.26)	0.34 (0.29, 0.40)
Model 2	0.18 (0.12, 0.24)	0.28 (0.21, 0.34)	0.08 (0.001, 0.14)	0.19 (0.10, 0.29)	0.08 (0.01, 0.15)	0.04 (−0.02, 0.11)	0.13 (0.06, 0.19)	0.12 (0.06, 0.19)	0.17 (0.10, 0.25)	0.40 (0.33, 0.46)
Model 3	0.18 (0.11, 0.24)	0.26 (0.19, 0.33)	0.07 (−0.01, 0.15)	0.20 (0.10, 0.30)	0.07 (−0.01, 0.14)	0.04 (−0.03, 0.11)	0.12 (0.05, 0.19)	0.10 (0.04, 0.17)	0.16 (0.08, 0.24)	0.42 (0.35, 0.49)
Sensitivity analysis	0.67 (0.58, 0.77)	0.66 (0.42, 0.90)	0.33 (0.21, 0.46)	0.61 (0.30, 0.91)	−0.01 (−0.26, 0.24)	−0.07 (−0.32, 0.19)	0.55 (0.33, 0.77)	0.40 (0.19, 0.61)	0.28 (0.04, 0.53)	1.37 (1.13, 1.60)

Abbreviations: BMI: body mass index; HDL: high density lipoprotein; LDL: low density lipoprotein; Numbers in the table from results of General Linear Regression model, with family being ‘binomial” and link “logit” for the association of dietary pattern with cardio metabolic risks; Model 1: Adjusted for age and sex; Model 2: model 1 + urbanization + income + education; Model 3: model 2 + smoking + drinking + physical activity; Sensitivity analysis: including participants with all rounds of food intake (*n* = 2318); Abdominal overweight/obesity defined as WC ≥ 94 cm in males and ≥80 cm in females; Hypertension defined as measured SBP ≥ 140 mmHg and/or SBP ≥ 90 mmHg and/or known diagnosis; Diabetes defined as fasting plasm glucose > 7.0 mmol/L, and/or diagnosed diabetes; High triglycerides defined as triglycerides ≥ 150 mg/dL; low HDL as HDL < 40 mg/dL in males or <50 mg/dL in females; high LDL defined as LDL ≥ 130 mg/dL; high cholesterol ≥ 200 mg/dL; Metabolic syndrome defined by IDF criteria; Impaired glucose control defined as HbA1C ≥ 5.7%.

## Supplementary Materials

The following are available online at www.mdpi.com/2072-6643/9/11/1218/s1, Figure S1: Age and sex adjusted “Traditional” score by urbanization (left panel) and education (right panel) among adults during 1991–2011, Table S1: Characteristics of adults in the China Health and Nutrition surveys during 1991–2011.
