# Supplementary material for: Dietary Pattern during 1991–2011 and Its Association with Cardio Metabolic Risks in Chinese Adults: The China Health and Nutrition Survey
Source: Nutrients. 2017 Nov 6;9(11):1218. doi: 10.3390/nu9111218 (PMC5707690; doi:10.3390/nu9111218)
Supplement: Supplementary file 1 [file nutrients-09-01218-s001.zip › Supplementary.docx]

**Table S1. Characteristics of adults in the China Health and Nutrition surveys during 1991-2011**

|  | **1991** | **1993** | **1997** | **2000** | **2004** | **2006** | **2009** | **2011** | ***p* for trend** |
| --- | --- | --- | --- | --- | --- | --- | --- | --- | --- |
| **N** | **11112** | **10816** | **11880** | **13312** | **13183** | **15911** | **16305** | **19721** |  |
| **Age (years)** | 40.1 (16.4) | 40.6 (16.5) | 41.8 (16.7) | 42.6 (16.6) | 44.3 (17.0) | 43.5 (16.8) | 44.5 (16.9) | 46.0 (16.6) | <0.001 |
| **Male (%)** | 5402 (48.6) | 5279 (48.8) | 5816 (50.0) | 6506 (48.9) | 6454 (49.0) | 7518 (47.3) | 7620 (46.7) | 9270 (47.0) | <0.001 |
| **Urbanization** |  |  |  |  |  |  |  |  | <0.001 |
| Low | 5678 (51.1) | 5210 (48.2) | 4601 (38.7) | 3991 (30.0) | 3781 (28.7) | 3641 (22.9) | 2498 (15.3) ) | 2,573 (13.1) |  |
| Medium | 3809 (34.3) | 3597 (33.3) | 3743 (31.5) | 4163 (31.1) | 3918 (29.7) | 5372 (33.8) | 6504 (39.9) | 6428 (32.8) |  |
| High | 1625 (14.6) | 2009 (18.6) | 3536 (29.8) | 5158 (38.8) | 5484 (41.6) | 6898 (43.4) | 7303 (44.8) | 10595 (54.1) |  |
| **Income** |  |  |  |  |  |  |  |  | <0.001 |
| Low | 3414 (30.8) | 3325 (31.0) | 3758 (31.9) | 4204 (32.2) | 4212 (32.3) | 5070 (32.5) | 5215 (32.5) | 6242 (32.2) |  |
| Medium | 3649 (32.9) | 3585 (33.4) | 3867 (32.8) | 4242 (32.5) | 4285 (32.8) | 5152 (33.0) | 5284 (32.9) | 6422 (33.1) |  |
| High | 4021 (36.3) | 3834 (35.7) | 4161 (35.3) | 4622 (35.4) | 4565 (35.0) | 5377 (34.5) | 5559 (34.6) | 6718 (34.7) |  |
| **Education** |  |  |  |  |  |  |  |  | <0.001 |
| Low | 5291 (54.1) | 4731 (51.5) | 4699 (49.1) | 4489 (44.2) | 4181 (42.5) | 4033 (41.3) | 4026 (40.0) | 4381 (33.7) |  |
| Medium | 2894 (29.6) | 2861 (31.1) | 2996 (31.3) | 3346 (33.0) | 3207 (32.6) | 3052 (31.3) | 3466 (34.5) | 4341 (33.4) |  |
| High | 1591 (16.3) | 1596 (17.4) | 1874 (19.6) | 2316 (22.8) | 2446 (24.9) | 2677 (27.4) | 2569 (25.5) | 4287 (33.0) |  |
| **BMI (Kg/m^2^)** | 21.7 (2.9) | 21.8 (2.9) | 22.3 (3.1) | 22.8 (3.3) | 23.1 (3.4) | 23.2 (3.4) | 23.3 (3.5) | 23.8 (3.7) | <0.001 |
| **SBP (mmHg)** | 114.8 (18.6) | 115.5 (17.8) | 118.9 (18.1) | 119.8 (18.0) | 122.4 (18.7) | 121.7 (18.0) | 124.6 (18.8) | 124.5 (17.7) | <0.001 |
| **DBP (mmHg)** | 74.4 (11.4) | 75.5 (11.2) | 77.2 911.0) | 77.7 (11.1) | 78.7 (12.3) | 78.9 (11.0) | 80.5 (11.3) | 79.3 (10.7) | <0.001 |
| **Smoking** |  |  |  |  |  |  |  |  | <0.001 |
| Never | 5649 (64.9) | 5441 (66.0) | 7111 (68.1) | 6598 (68.5) | 6624 (67.3) | 6697 (68.5) | 6915 (68.7) | 9039 (69.3) |  |
| Previous | 192 (2.2) | 143 (1.7) | 131 (1.3) | 152 (1.6) | 366 (3.7) | 425 (4.3) | 343 (3.4) | 565 (4.3) |  |
| Current | 2862 (32.9) | 2657 (32.2) | 3201 (30.7) | 2886 (30.0) | 2850 (29.0) | 2660 (27.2) | 2815 (28.0) | 3431 (26.3) |  |
| **Drinking** |  |  |  |  |  |  |  |  | <0.001 |
| Never | 5406 (63.0) | 5308 (64.6) | 6569 (64.6) | 6125 (65.5) | 6595 (67.7) | 6666 (68.8) | 6724 (67.2) | 8615 (66.4) |  |
| <1/week | 868 (14.2) | 838 (10.2) | 1118 (11.0) | 862 (9.2) | 1221 (8.9) | 775 (8.0) | 1178 (11.8) | 1586 (12.2) |  |
| 1-2/week | 711 (8.3) | 766 (9.3) | 914 (9.0) | 835 (8.9) | 813 (8.3) | 857 (8.8) | 771 (7.7) | 975 (7.5) |  |
| 3-4/week | 419 (4.9) | 444 (5.4) | 564 (5.6) | 511 (5.5) | 425 (4.4) | 401 (4.1) | 426 (4.3) | 501 (3.9) |  |
| Daily | 819 (9.6) | 858 (10.5) | 998 (9.8) | 1021 (10.9) | 1043 (10.7) | 997 (10.3) | 913 (9.1) | 1291 (10.0) |  |
| **Median METs (IQR)** | 17.9 (7.4, 185.2) | 15.8 (5.2, 136.3) | 8.0 (5.0, 16.34) | 8.6 (5.0, 17.1) | 51.3 (13.3, 159.6) | 49.5 (13.3, 160.6) | 70.4 (27.7, 163.7) | 74.0 (29.9, 148.8) | <0.001 |

Abbreviations: SBP: systolic blood pressure; DBP: diastolic blood pressure; METs: metabolic equivalent task score; IQR: interquartile range.

Urbanization defined by a twelve-component urbanization index including capture population density and physical, social, cultural and economic environments (reference)

Income defined by per capita annual family income.

**
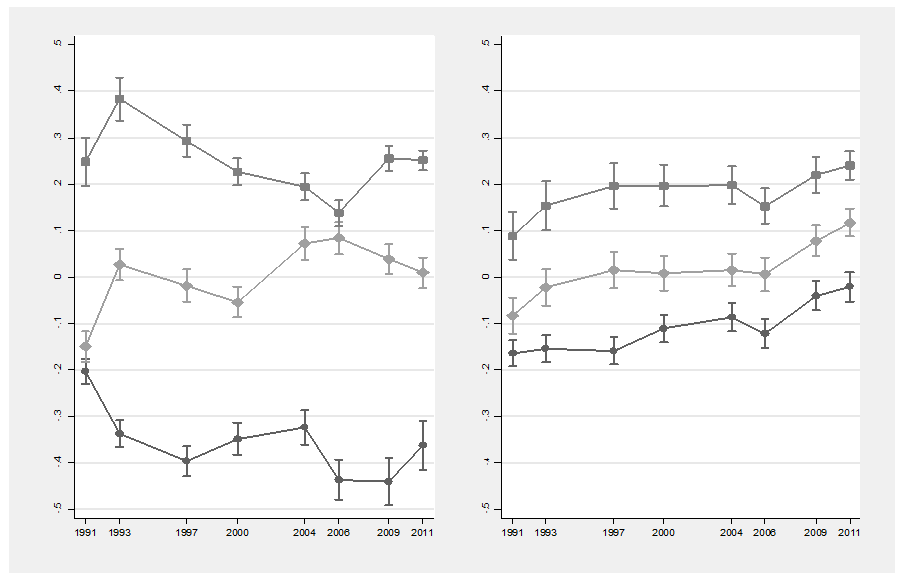
**

**Figure S1. Age and sex adjusted “Traditional” score by urbanization (left panel) and education (right panel) among adults during 1991-2011**


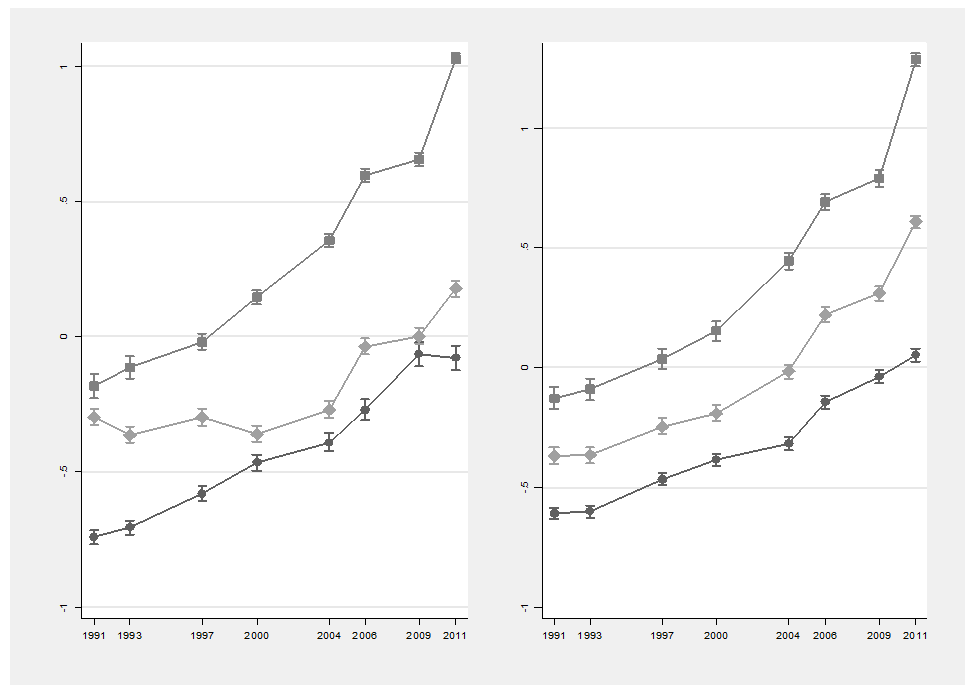


**Supplement Figure 2. Age and sex adjusted “Modern” score positively associated with urbanization (left panel) and education (right panel) among adults** **during 1991-2011**
